# An SEIV Epidemic Model for Childhood Diseases with Partial Permanent Immunity

**DOI:** 10.1155/2015/420952

**Published:** 2015-05-18

**Authors:** Mei Bai, Lishun Ren

**Affiliations:** ^1^Department of Mathematics and Statistics, Zhoukou Normal University, Zhoukou, Henan 466000, China; ^2^Department of Mathematics and Statistics, Zhengzhou University, Zhengzhou, Henan 450001, China

## Abstract

An SEIV epidemic model for childhood disease with partial permanent immunity is studied. The basic reproduction number *R*_0_ has been worked out. The local and global asymptotical stability analysis of the equilibria are performed, respectively. Furthermore, if we take the treated rate *τ* as the bifurcation parameter, periodic orbits will bifurcate from endemic equilibrium when *τ* passes through a critical value. Finally, some numerical simulations are given to support our analytic results.

## 1. Introduction

It is primarily important for health administrators to protect children from disease that can be prevented by vaccination. Although preventive vaccines have reduced the incidence of infectious diseases among children, childhood disease is an important public health problem. We often use mathematical models to realize the transmission dynamics of childhood diseases and to estimate control programs [1–4]. Recently, many scholars study the SEIV epidemic models [5, 6]. In those models, let *S*(*t*), *I*(*t*), and *V*(*t*), respectively, represent the number of susceptible individuals at time *t*, infective individuals at time *t*, and vaccinated individuals at time *t*. At the earliest, most researches on these types of models assume that the disease incubation is negligible, so that each susceptible individual, once infected, instantaneously turns into infectious and later recovers obtaining a permanent immunity. Soon afterwards, the models become more general. Researchers assume that a susceptible individual first goes through a latent period after infection before becoming infectious (we called *E* represents exposed individuals but not yet infectious).

In [7], the authors discussed the following model:

(1) S˙(t)=A(1−p)+ωV−μS−βSI,V˙(t)=Ap−ωV−μV+τI,E˙(t)=βSI−μE−σE,I˙(t)=σE−μI−τI,

where all parameters are positive. Parameter *A* represents the number of additional populations of childhood; *ω* represents the rate at which vaccine wanes; *μ* represents the natural death rate; *β* represents the rate at which susceptible individuals become infected by those who are infectious; *p* represents the fraction of recruited individuals who are vaccinated; *τ* represents the rate at which infected individuals are treated; and *σ* represents the rate at which exposed individuals become infectious.

In model (1), *βSI* is called incidence rate which plays an important role in the transmission dynamics. In addition, incidence rate can determine the tendency of epidemics. At the earliest, in the classical epidemic disease model, scholars made much focus on the bilinear incidence [8, 9]. In 1945, Wilson and Worcester discussed the nonlinear incidence rate [10, 11]. Later, the incidence function grows into more general nonlinear forms. In [12], the authors have considered a SEIV model with nonlinear incidence rate *βSI*(1 + *αI*). The paper discussed the basic reproduction of the system and bifurcation phenomenon. And this incidence function is more in line with actual situation. One of the strategies to control infectious diseases is vaccination in [13, 14].

And under the above circumstance, in [15], the authors have studied the following model:

(2) S˙(t)=A(1−p)+ωV−μS−βSI(1+αI),V˙(t)=Ap−ωV−μV+τI,E˙(t)=βSI(1+αI)−μE−σE,I˙(t)=σE−μI−τI.

In [15] they have supplied a framework of discussing the transmission dynamics of the epidemic model where the preventive vaccine may lose efficacy over time. And it has showed that if the vaccination coverage level is below the threshold, the disease will persist within the population. In addition, if the vaccination coverage level exceeds a certain threshold value, the disease can be eradicated from the population through constructing a proper Lyapunov function by using global stability analysis of the model.

In the process of treatment, some patients can not be cured; therefore we should consider the disease-caused death on the basic of the above models and make the parameter *ϵ* be the rate at which infectious individuals lose their life due to disease during the process of treatment. Moreover, about some diseases, some cured patients can not obtain a permanent immunity. Thus, this paper also considers the SEIV epidemic models for childhood disease with partial permanent immunity based on above models and denotes *τ* as the rate of *I* transforming to *S*. Namely, when *τ* = 0, all recoverers obtain permanent immunity. When *τ* = 1, all recoverers become susceptible individuals. When 0 < *τ* < 1, partial infective individuals become susceptible individuals and the number is *τI*. So model (2) is transformed to model (3). Model (3) is described as follows:

(3) S˙(t)=(1−p)A+ωV+τI−μS−βSI(1+αI),V˙(t)=pA−ωV−μV,E˙(t)=βSI(1+αI)−μE−σE,I˙(t)=σE−μI−τI−ϵI.

Assume the initial values are satisfied with the following:

(4) $$\begin{array}{c} S\left(0\right)\geq 0,\;\;V\left(0\right)\geq 0,\\ E\left(0\right)\geq 0,\;\;I\left(0\right)\geq 0. \end{array}$$

System (3) which we present will be analyzed to decide the optimal vaccine coverage level needed to control the disease. The rest of this paper is organized as follows. In Section 2, we calculate the basic reproduction number *R*_0_, which determines the spread of infection. In Section 3, the local stability of equilibria is analyzed. We discuss the bifurcation phenomenon and illustrate that when the treated rate *τ* crosses through a critical value, system (3) undergoes Hopf bifurcation at the positive equilibrium in Section 4. By constructing the Lyapunov function and a generalization of the Poincaré-Bendixson criterion, we discuss the global stability of disease-free equilibrium and endemic equilibrium, respectively, in Section 5. Some numerical examples are presented to illustrate theoretical analysis in Section 6. In Section 7 we discuss our findings.

## 2. The Basic Reproduction Number

In the following, we will calculate the basic reproduction number of system (3). The basic reproduction number, denoted by *R*_0_, is the expected number of secondary cases produced, in a completely susceptible population, by a typical infective individual [16]. Obviously, system (3) always has a disease-free equilibrium *P*_0_(*A*[*μ*(1 − *p*) + *ω*]/*μ*(*μ* + *ω*), *pA*/(*μ* + *ω*), 0,0); that is, *E* = *I* = 0. And {(*S*, *E*, *I*, *V*)∣*S* > 0, *E* ≥ 0, *I* ≥ 0, *V* > 0} is a positively invariant set of system (3). Adding up the four equations in system (3), we can obtain

(5) ddtS+E+I+V=A−μ(S+E+I+V)−ϵI=μ[Aμ−(S+E+I+V)]−ϵI≤μ[Aμ−(S+E+I+V)].

And limsup⁡_*t*→*∞*_⁡(*S* + *E* + *I* + *V*) ≤ *A*/*μ*. Therefore, the set

(6) Ω=(S,E,I,V) ∣ S>0,  E≥0,  I≥0,  V>0,AμmmcS+E+I+V≤Aμ

is positively invariant for system (3). Next we will discuss the dynamic characteristic of system (3) on *Ω*. Set *x* = (*E*, *I*, *S*, *V*)^*T*^; then system (3) can be rewritten as

(7) $$\begin{array}{c} \frac{dx}{dt}=\Phi \left(x\right)-\Psi \left(x\right), \end{array}$$

where

(8) $$\begin{array}{c} \Phi \left(x\right)=\left[\begin{array}{c} \beta SI\left(1+\alpha I\right)\\ 0\\ 0\\ 0 \end{array}\right],\\ \Psi \left(x\right)=\left[\begin{array}{c} \mu E+\sigma E\\ \mu I+\tau I+\epsilon I-\sigma E\\ -A\left(1-p\right)-\omega V-\tau I+\mu S+\beta SI\left(1+\alpha I\right)\\ -pA+\omega V+\mu V \end{array}\right]. \end{array}$$

Define

(9) $$\begin{array}{c} F=\left[\frac{\partial \Phi_{i}}{\partial x_{j}}\left(x_{0}\right)\right],\;V=\left[\frac{\partial \Psi_{i}}{\partial x_{j}}\left(x_{0}\right)\right]\;\text{with}\;\;1\leq i,j\leq 2. \end{array}$$

We have

(10) $$\begin{array}{c} F=\left[\begin{array}{cc} 0 & \frac{\beta A\left[\mu \left(1-p\right)+\omega \right]}{\mu \left(\mu +\omega \right)}\\ 0 & 0 \end{array}\right],\\ V=\left[\begin{array}{cc} \mu +\sigma & 0\\ -\sigma & \mu +\tau +\epsilon \end{array}\right]. \end{array}$$

It is easy to get

(11) $$\begin{array}{c} V^{-1}=\left[\begin{array}{cc} \frac{1}{\mu +\sigma } & 0\\ \frac{\sigma }{\left(\mu +\sigma \right)\left(\mu +\tau +\epsilon \right)} & \frac{1}{\mu +\tau +\epsilon } \end{array}\right]. \end{array}$$

*FV* ^−1^ develops a meaningful definition of *R*_0_ and is the expected number of new infections for system (3). *ρ*(*FV* ^−1^) = *σβA*[*μ*(1 − *p*) + *ω*]/*μ*(*μ* + *ω*)(*μ* + *σ*)(*μ* + *τ* + *ϵ*) is the spectral radius of matrix *FV* ^−1^. Thus by [16]

(12) $$\begin{array}{c} R_{0}=\frac{\sigma \beta A\left[\mu \left(1-p\right)+\omega \right]}{\mu \left(\mu +\omega \right)\left(\mu +\sigma \right)\left(\mu +\tau +\epsilon \right)}. \end{array}$$

Set

(13) R0∗=1αμM ·[2αμMβ[M−(μ+ω)στ]−β[M−στ(μ+ω)]],R1∗=β[M−τσ(ω+μ)]αμM,

where *M* = (*μ* + *ω*)(*μ* + *σ*)(*μ* + *τ* + *ϵ*).

**Lemma 1 .**

Assume that *R*_0_, *R*_0_^*^, and *R*_1_^*^ are defined as (12),  (13), and *M* > *στ*(*μ* + *ω*); then

- *R*_0_^*^ ≤ 1;
- *R*_1_^*^ < 1⇔*R*_1_^*^ < *R*_0_^*^.

**Proof**

(i) From the definition of *R*_0_^*^, we know

(14) R0∗−1=2αμMβ[M−(μ+ω)στ]mmcαμMβ[M−(μ+ω)στ]−βM−στμ+ω−αμMαμM−1,

for *M* > *στ*(*μ* + *ω*) and *α*, *β*, *μ*, *M* > 0; then

(15) $$\begin{array}{c} \beta \left[M-\sigma \tau \left(\mu +\omega \right)\right]\geq 0,\;\;\alpha \mu M\geq 0,\\ \beta \left[M-\sigma \tau \left(\mu +\omega \right)\right]+\alpha \mu M\geq 2\sqrt{\alpha \mu M\beta \left[M-\left(\mu +\omega \right)\sigma \tau \right]}; \end{array}$$

thus

(16) $$\begin{array}{c} R_{0}^{*}-1\leq 0; \end{array}$$

that is

(17) $$\begin{array}{c} R_{0}^{*}\leq 1. \end{array}$$

(ii) From the definition of *R*_1_^*^, we know

(18) R1∗<1⟺R1∗−1=β[M−τσ(ω+μ)]−αμMαμM<0⟺β[M−τσ(ω+μ)]<αμM,R1∗<R0∗⟺R1∗−R0∗=2βM−τσω+μ−2αμMβM−μ+ωσταμM<0⟺β[M−τσ(ω+μ)]<αμMβ[M−(μ+ω)στ]⟺β[M−τσ(ω+μ)]<αμM;

this completes the proof.

## 3. Local Stability of Equilibria

In the following, we will discuss the local stability of the equilibria *P*_0_ and *P* ^*^.

**Theorem 2 (see [16]).**

The disease-free equilibrium *P*_0_ is locally asymptotically stable if *R*_0_ < 1; it is unstable if *R*_0_ > 1.

**Theorem 3 .**

(i) Suppose *R*_1_^*^ < 1. When *R*_0_ < *R*_0_^*^ system (3) has no real equilibria; when *R*_0_^*^ < *R*_0_ < 1 there are two endemic equilibria, *P*_1_^*^ and *P*_2_^*^, and when *R*_0_ ≥ 1 there is only one endemic equilibrium *P* ^*^.

(ii) Suppose *R*_1_^*^ > 1. When *R*_0_ < *R*_0_^*^ system (3) has no real equilibria; when *R*_0_^*^ < *R*_0_ < 1 there is no endemic equilibria, and when *R*_0_ ≥ 1 there is only one unique endemic equilibrium *P* ^*^.

**Proof**

Through the following system, we can calculate the endemic equilibria *P* ^*^(*S* ^*^, *V* ^*^, *E* ^*^, *I* ^*^):

(19) $$\begin{array}{c} A\left(1-p\right)+\omega V^{*}+\tau I^{*}-\mu S^{*}-\beta S^{*}I^{*}\left(1+\alpha I^{*}\right)=0,\\ Ap-\omega V^{*}-\mu V^{*}=0,\\ \beta S^{*}I^{*}\left(1+\alpha I^{*}\right)-\mu E^{*}-\sigma E^{*}=0,\\ \sigma E^{*}-\mu I^{*}-\tau I^{*}-\epsilon I^{*}=0. \end{array}$$

We can get

(20) E∗=(μ+τ+ϵσ)I∗,S∗=(μ+σ)(μ+τ+ϵ)σβ(1+αI∗),V∗=pAμ+ω.

*I* ^*^ is satisfied with the following equation and is positive:

(21) $$\begin{array}{c} k_{1}{\left(I^{*}\right)}^{2}+k_{2}I^{*}+k_{3}=0, \end{array}$$

where

(22) k1=αβ[(μ+ω)στ−M],k2=M(μαR0−β)+σβτ(μ+ω),k3=μM(R0−1).

We have

(23) $$\begin{array}{c} k_{1}<0,\;k_{2}>0⟺R_{0}>R_{1}^{*};\\ k_{3}>0⟺R_{0}>1; \end{array}$$

it indicates the case of equilibria for system (3). More specifically, when *k*_3_ > 0 system (3) has only one endemic equilibrium; when *k*_3_ < 0, *k*_2_ > 0, and (*k*_2_)^2^ − 4*k*_1_ *k*_3_ > 0 it has two endemic equilibria; otherwise it has no endemic equilibria by the Descartes rule of signs. And, for *k*_3_ < 0, *k*_2_ > 0, and (*k*_2_)^2^ − 4*k*_1_ *k*_3_ = 0, that is, when *R*_0_ = *R*_0_^*^, we notice there exists a bifurcation point. Actually, the formula *k*_2_^2^ − 4*k*_1_ *k*_3_ can be represented with respect to *R*_0_ so that

(24) k22−4k1k3=μMαR0+βM−στμ+ω2 −4μMαβ[M−στ(μ+ω)].

Hence, (*k*_2_)^2^ − 4*k*_1_ *k*_3_ ≥ 0 when *R*_0_ ≥ *R*_0_^*^. Considering all the analysis results, (i) and (ii) can be obtained easily.

**Theorem 4 .**

For *R*_0_ > 1, the endemic equilibrium *P* ^*^ of system (3) is locally asymptotically stable satisfying *c*_3_ > 0 and *c*_1_ *c*_2_ − *c*_3_ > 0, where *c*_1_, *c*_2_, and *c*_3_ are shown in the following proof.

**Proof**

System (3) has only one endemic equilibrium *P* ^*^ for *R*_0_ > 1. At the equilibrium *P* ^*^ = (*S* ^*^, *V* ^*^, *E* ^*^, *I* ^*^) the matrix of the linearized system (3) is

(25) $$\begin{array}{c} J\left(P^{*}\right)=\left[\begin{array}{cccc} -\mu -\beta I^{*}\left(1+\alpha I^{*}\right) & \omega & 0 & \tau -\beta S^{*}\left(1+2\alpha I^{*}\right)\\ 0 & -\omega -\mu & 0 & 0\\ \beta I^{*}\left(1+\alpha I^{*}\right) & 0 & -\mu -\sigma & \beta S^{*}\left(1+2\alpha I^{*}\right)\\ 0 & 0 & \sigma & -\tau -\mu -\epsilon \end{array}\right]. \end{array}$$

The characteristic equation is

(26) $$\begin{array}{c} \left(\lambda +\omega +\mu \right)\left(\lambda^{3}+c_{1}\lambda^{2}+c_{2}\lambda +c_{3}\right)=0, \end{array}$$

where

(27) c1=(μ+E)+(2μ+τ+σ+ϵ)=3μ+τ+σ+ϵ+E,c2=(μ+E)(2μ+τ+σ+ϵ)+(τ+μ+ϵ)(μ+σ)−σF,c3=(μ+E)(τ+μ+ϵ)(μ+σ)+στE−μσF,E=βI∗(1+αI∗),F=βS∗(1+2αI∗).

It is easy to get

(28) c1c2−c3=(2μ+σ+E)τ2 +μ+E5μ+2σ+2ϵ+Emmmc+(μ+σ)(4μ+σ+2ϵ+E)mmmc−σF−(μ+E)(μ+σ)−σEτ +(μ+E)(σ+ϵ+E+3μ)(2μ+σ+ϵ) +(μ+σ)(3μ+σ+ϵ+E)(μ+ϵ) −σF(3μ+σ+ϵ+E) −(μ+E)(μ+σ)(μ+ϵ)+μσF=Dτ2+Gτ+H.

It is clear that *c*_1_ > 0. By the Hurwitz criterion, epidemic equilibrium *P* ^*^ is locally asymptotically stable for *c*_3_ > 0 and *c*_1_ *c*_2_ − *c*_3_ > 0.

## 4. Bifurcation Analysis

From Theorem 3 we can see that *R*_0_ = 1 is a bifurcation value. Actually, the disease-free equilibrium changes its stability when being across *R*_0_ = 1. Next, we investigate the nature of the bifurcation concerning the disease-free equilibrium *P*_0_(*A*[*μ*(1 − *p*) + *ω*]/*μ*(*μ* + *ω*), *pA*/(*μ* + *ω*), 0,0) when *R*_0_ = 1. In other words, we will discuss under what conditions system (3) can undergo a forward or a backward bifurcation. And we need the results in [17, 18]. In order to introduce it, consider the following equation which has a parameter *ϕ*:

(29) $$\begin{array}{c} \frac{dx}{dt}=f\left(x,\phi \right);\;f:R^{n}\times R^{n},\;f\in C^{2}\left(R^{n}\times R^{n}\right). \end{array}$$

Without loss of generality, for all values of the parameter *ϕ*, assume 0 is an equilibrium for system (29); that is,

(30) $$\begin{array}{c} f\left(0,\phi \right)\equiv 0,\;\forall \phi =0. \end{array}$$

**Lemma 5 (see [17]).**

Suppose the following.

(A1) *Q* = *D*_*x*_ *f*(0,0) is the linearization matrix of system (29) around the equilibrium *x* = 0 with *ϕ* evaluated at 0. 0 is a simple eigenvalue of *Q* and all other eigenvalues of *Q* have negative real parts.
(A2) Matrix *Q* has a (nonnegative) right eigenvector *V* and a left eigenvector *W* with respect to the zero eigenvalue.

Define *f*_*k*_ as the *k*th component of *f*, and

(31) a=∑k,i,j=1nwkvivj∂2fk∂xi∂xj(0,0),b=∑k,i=1nwkvi∂2fk∂xi∂ϕ(0,0).

And *a* and *b* totally decide the local dynamic of system (29) around *x* = 0.

- Consider *a* > 0, *b* > 0. If *ϕ* < 0, with |*ϕ* | ≪1, *x* = 0 is locally asymptotically stable and there exists a positive unstable equilibrium; if 0 < *ϕ* ≤ 1, *x* = 0 is unstable and there exists a negative and locally asymptotically stable equilibrium.
- Consider *a* < 0, *b* < 0. If *ϕ* < 0, with |*ϕ* | ≪1, *x* = 0 is unstable; if 0 < *ϕ* ≤ 1, *x* = 0 is locally asymptotically stable and there exists a positive unstable equilibrium.
- Consider *a* > 0, *b* < 0. If *ϕ* < 0, with |*ϕ* | ≪1, *x* = 0 is unstable and there exists a locally asymptotically stable negative equilibrium; if 0 < *ϕ* ≤ 1, *x* = 0 is stable and a positive unstable equilibrium emerges.
- Consider *a* < 0, *b* > 0. If *ϕ* changes from negative to positive, *x* = 0 changes its stability from stable to unstable. Correspondingly, a negative unstable equilibrium becomes positive and locally asymptotically stable.

**Remark 6 .**

The requirement that *V* is nonnegative is unnecessary by [17].

It seems that a transcritical bifurcation occurs at *ϕ* = 0: more specifically, the bifurcation at *ϕ* = 0 is forward when *a* < 0 and *b* > 0; the bifurcation at *ϕ* = 0 is backward when *a* > 0 and *b* > 0.

Next consider *ϕ* = 0 as the bifurcation parameter, so that *R*_0_ < 1 for *ϕ* < 0 and *R*_0_ > 1 for *ϕ* > 0 and so that *x*_0_ is a disease-free equilibrium for system (29) of all values of *ϕ*.

Take into account the following system:

(32) $$\begin{array}{c} \frac{dx}{dt}=f\left(x,\phi \right), \end{array}$$

where *f* is continuously differentiable at least twice in both *x* and *ϕ*. The disease-free equilibrium is the line (*x*_0_; *ϕ*). And the disease-free equilibrium changes its local stability at the point (*x*_0_; *ϕ*) [16].

Next we will exhibit that there exist nontrivial equilibria near the bifurcation point (*x*_0_; 0).

Let *S* = *x*_1_, *V* = *x*_2_, *E* = *x*_3_, and *I* = *x*_4_; then system (3) becomes

(33) x1˙t=A(1−p)+ωx2(t)+τx4(t)−μx1(t) −βx1(t)x4(t)(1+αx4(t))≔f1,x2˙(t)=Ap−ωx2(t)−μx2(t)≔f2,x3˙(t)=βx1(t)x4(t)(1+αx4(t))−μx3(t)−σx3(t)≔f3,x4˙(t)=σx3(t)−μx4(t)−τx4(t)−ϵx4(t)≔f4.

We will show that system (33) may exhibit a backward bifurcation when *R*_0_ = 1 by applying Lemma 5. Think of the disease-free equilibrium *P*_0_(*A*[*μ*(1 − *p*) + *ω*]/*μ*(*μ* + *ω*), *pA*/(*μ* + *ω*), 0,0) and notice that the condition *R*_0_ = 1 can be seen as *β* = *β* ^*^ = *μ*(*μ* + *τ* + *ϵ*)(*μ* + *σ*)(*μ* + *ω*)/*σA*[(1 − *p*)*μ* + *ω*] in terms of the parameter *β*.

Calculate the eigenvalues of the following matrix:

(34) J(P0,β∗) =−μω0τ−β∗A[μ(1−p)+ω]μ(μ+ω)0−ω−μ0000−μ−σβ∗A[μ(1−p)+ω]μ(μ+ω)00σ−τ−μ−ϵ;

we can obtain *λ*_1_ = −*μ*, *λ*_2_ = −*μ* − *ω*, *λ*_3_ = −2*μ* − *σ* − *τ*, and *λ*_4_ = 0.

The matrix *J*(*P*_0_, *β* ^*^) has a simple eigenvalue of 0; and all others have negative real parts. Thus, we can make use of the center manifold theory. The disease-free equilibrium *P*_0_ is a nonhyperbolic equilibrium when *β* = *β* ^*^ (i.e., when *R*_0_ = 1). This completes the verification with respect to (A1) of Lemma 5.

Now we set *V* = (*v*_1_, *v*_2_, *v*_3_, *v*_4_)^*T*^ as a right eigenvector associated with the zero eigenvalue *λ*_4_ = 0. It is calculated by

(35) $$\begin{array}{c} \left[\begin{array}{cccc} -\mu & \omega & 0 & \tau -\frac{\beta^{*}A\left[\mu \left(1-p\right)+\omega \right]}{\mu \left(\mu +\omega \right)}\\ 0 & -\omega -\mu & 0 & 0\\ 0 & 0 & -\mu -\sigma & \frac{\beta^{*}A\left[\mu \left(1-p\right)+\omega \right]}{\mu \left(\mu +\omega \right)}\\ 0 & 0 & \sigma & -\tau -\mu -\epsilon \end{array}\right]\left[\begin{array}{c} v_{1}\\ v_{2}\\ v_{3}\\ v_{4} \end{array}\right]=0. \end{array}$$

Expanding (35), we can have

(36) $$\begin{array}{c} -\mu v_{1}+\omega v_{2}+\tau v_{4}-\frac{\beta^{*}A\left[\mu \left(1-p\right)+\omega \right]}{\mu \left(\mu +\omega \right)}v_{4}=0,\\ \left(-\omega -\mu \right)v_{2}=0,\\ \left(-\mu -\sigma \right)v_{3}+\frac{\beta^{*}A\left[\mu \left(1-p\right)+\omega \right]}{\mu \left(\mu +\omega \right)}v_{4}=0,\\ \sigma v_{3}-\left(\tau +\mu +\sigma \right)v_{4}=0. \end{array}$$

Expanding (36), we have

(37) $$\begin{array}{c} V={\left(\frac{\left(\mu +\sigma )[\sigma \tau (\mu +\omega )-M\right]}{\mu M},0,1,\frac{\sigma }{\tau +\mu +\epsilon }\right)}^{T}. \end{array}$$

And the left eigenvector *W* = (*w*_1_, *w*_2_, *w*_3_, *w*_4_) satisfying *W* · *V* = 1 is obtained by

(38) −μw1=0,ωw1−(ω+μ)w2=0,−(μ+σ)w3+σw4=0,τw1−β∗A[μ(1−p)+ω]μ(μ+ω)w1+β∗A[μ(1−p)+ω]μ(μ+ω)w3  −(τ+μ+ϵ)w4=0.

From (38), the left eigenvector *W* turns out to be

(39) $$\begin{array}{c} W={\left(0,0,\frac{\left(\mu +\tau +\epsilon \right)}{2\mu +\sigma +\tau +\epsilon },\frac{\left(\mu +\sigma )(\mu +\tau +\epsilon \right)}{\sigma (2\mu +\sigma +\tau +\epsilon )}\right)}^{T}. \end{array}$$

Computing the following formulas, we get

(40) $$\begin{array}{c} \frac{\partial^{2}f_{1}}{\partial x_{1}\partial x_{4}}=\frac{\partial^{2}f_{1}}{\partial x_{4}\partial x_{1}}=-\beta ,\\ \frac{\partial^{2}f_{1}}{\partial x_{4}^{2}}=\frac{-2\alpha \beta A\left[\mu \left(1-p\right)+\omega \right]}{\mu \left(\mu +\omega \right)},\\ \frac{\partial^{2}f_{3}}{\partial x_{1}\partial x_{4}}=\frac{\partial^{2}f_{3}}{\partial x_{4}\partial x_{1}}=\beta ,\\ \frac{\partial^{2}f_{3}}{\partial x_{4}^{2}}=\frac{2\alpha \beta A\left[\mu \left(1-p\right)+\omega \right]}{\mu \left(\mu +\omega \right)},\\ \frac{\partial^{2}f_{1}}{\partial x_{4}\partial \beta }=\frac{\partial^{2}f_{1}}{\partial \beta \partial x_{4}}=\frac{-A\left[\mu \left(1-p\right)+\omega \right]}{\mu \left(\mu +\omega \right)},\\ \frac{\partial^{2}f_{3}}{\partial x_{4}\partial \beta }=\frac{\partial^{2}f_{3}}{\partial \beta \partial x_{4}}=\frac{-A\left[\mu \left(1-p\right)+\omega \right]}{\mu \left(\mu +\omega \right)}, \end{array}$$

and all the other second-order partial derivatives are equal to zero.

So, we evaluate *a* and *b* as follows:

(41) $$\begin{array}{c} a=\overset{4}{\underset{k,i,j=1}{\sum }}w_{k}v_{i}v_{j}\frac{\partial^{2}f_{k}}{\partial x_{i}\partial x_{j}}\left(P_{0},\beta^{*}\right),\\ b=\overset{4}{\underset{k,i=1}{\sum }}w_{k}v_{i}\frac{\partial^{2}f_{k}}{\partial x_{i}\partial \beta }\left(P_{0},\beta^{*}\right). \end{array}$$

From system (33), and the terms (∂^2^ *f*_*k*_/∂*x*_*i*_∂*x*_*j*_)(*P*_0_, *β* ^*^) and (∂^2^ *f*_*k*_/∂*x*_*i*_∂*β*)(*P*_0_, *β* ^*^) which are nonzero, the following are deduced:

(42) a=2w1v1v4∂2f1∂x1∂x4(P0,β∗)+w1v42∂2f1∂x42(P0,β∗) +2w3v1v4∂2f3∂x1∂x4(P0,β∗)+w3v42∂2f3∂x42(P0,β∗),b=2w1v4∂2f1∂x4∂β(P0,β∗)+2w3v4∂2f3∂x4∂β(P0,β∗).

By (37) and (39), we obtain

(43) a=2σβμ(2μ+σ+τ+ϵ)(μ+σ)[στ(μ+ω)−M] +(μ+σ)σαA[μ(1−p)+ω],b=2σA[μ(1−p)+ω]μ(2μ+σ+τ+ϵ)(μ+ω).

Obviously *b* is always positive. Therefore the sign of the coefficient *a* determines the local dynamics around the disease-free equilibrium for *β* = *β* ^*^ by Lemma 5.

**Remark 7 .**

Set *α*_0_ = (*M* − *στ*(*μ* + *ω*))/*σA*[*μ*(1 − *p*) + *ω*]. The coefficient *a* is positive if and only if *α* > *α*_0_. Under this circumstance, the direction of the bifurcation for system (3) at *R*_0_ = 1 is backward. Considering condition (12), we get that the condition *α* > *α*_0_ is equivalent to the condition *R*_1_^*^ < 1 at the bifurcation, that is, when *R*_0_ = 1.

**Theorem 8 .**

Let *R*_0_ = 1. System (3) shows a backward bifurcation when *R*_1_^*^ < 1 and a forward bifurcation when *R*_1_^*^ > 1.

Furthermore, taking the treated rate *τ* as the bifurcation parameter, we can get the following.

**Theorem 9 .**

Let *R*_0_ > 1. When *τ* passes through a critical value, system (3) undergoes Hopf bifurcation at the positive equilibrium *P* ^*^.

**Proof**

If system (3) shows Hopf bifurcation, there must exist *τ* = *τ* ^*^, which satisfies the following conditions:

(44)  i h(τ∗)≡c1(τ∗)c2(τ∗)−c3(τ∗)=0,

(45)  ii ddτReλττ=τ∗≠0.

From (28) and (44), we can calculate the critical value *τ* ^*^.

For *τ* = *τ* ^*^, we have

(46) $$\begin{array}{c} c_{1}c_{2}=c_{3}. \end{array}$$

From (26) and (46), we have

(47) $$\begin{array}{c} \left(\lambda^{2}+c_{2}\right)\left(\lambda +c_{1}\right)=0, \end{array}$$

which has three roots:

(48) $$\begin{array}{c} \lambda_{1}=i\sqrt{c_{2}},\;\;\lambda_{2}=-i\sqrt{c_{2}},\;\;\lambda_{3}=-\sqrt{c_{1}}. \end{array}$$

For all *τ*, the roots are all in the following general forms:

(49) λ1(τ)=α1(τ)+iα2(τ),λ2(τ)=α1(τ)−iα2(τ),λ3(τ)=−c1.

Next, we prove the transversality condition

(50) ddτReλjττ=τ∗≠0, j=1,2.

We substitute *λ*_*j*_(*τ*) = *α*_1_(*τ*) + *iα*_2_(*τ*) into (47) and calculate the derivative, getting

(51) $$\begin{array}{c} J_{1}\left(\tau \right)\alpha_{1}^{\prime }\left(\tau \right)-G_{1}\left(\tau \right)\alpha_{2}^{\prime }\left(\tau \right)+K_{1}\left(\tau \right)=0,\\ G_{1}\left(\tau \right)\alpha_{1}^{\prime }\left(\tau \right)+J_{1}\left(\tau \right)\alpha_{2}^{\prime }\left(\tau \right)+H_{1}\left(\tau \right)=0, \end{array}$$

where

(52) G1=6α1(τ)α2(τ)+2c1(τ)α2(τ),H1=2α1(τ)α2(τ)c1′(τ)+c2′(τ)α2(τ),J1=3α12(τ)+2c1(τ)α1(τ)+c2(τ)−3α22(τ),K1=α12(τ)c1′(τ)+c2′(τ)α1(τ)+c3′(τ)−c1′(τ)α22(τ).

For

(53) $$\begin{array}{c} G_{1}\left(\tau^{*}\right)H_{1}\left(\tau^{*}\right)+J_{1}\left(\tau^{*}\right)K_{1}\left(\tau^{*}\right)\neq 0, \end{array}$$

we obtain

(54) ddτReλjττ=τ∗=G1H1+J1K1J12+G12τ=τ∗≠0.

Hence, the transversality condition is confirmed. This verifies the result.

## 5. Global Stability of Equilibria

**Theorem 10 .**

Let *R*_0_(1 + *αA*/*μ*) < 1. If *R*_1_^*^ > 1 the disease-free equilibrium *P*_0_ is globally asymptotically stable in *Ω*; if *R*_1_^*^ < 1 the disease-free equilibrium *P*_0_ is globally asymptotically stable in *Ω* when *R*_0_ < *R*_0_^*^.

**Proof**

When *R*_0_(1 + *αA*/*μ*) < 1, *R*_0_ < 1. If *R*_1_^*^ > 1, *P*_0_ is the only equilibrium of  (3) which is located in *Ω*. From the first equation of  (3), we obtain *dS*/*dt* ≤ (1 − *p*)*A* + (*A*/*μ*)(*ω* + *τ*)−(*μ* + *ω*)*S*. A solution of the equation *dy*/*dt* = (1 − *p*)*A* + (*A*/*μ*)(*ω* + *τ*)−(*μ* + *ω*)*y* is a upper solution of *S*(*t*). Due to that *y* → *A*[*μ*(1 − *p*)+(*ω* + *τ*)]/*μ*(*μ* + *ω*) when *t* → *∞*, we can easily get that, for a small enough *ε* > 0 which is sufficiently small, there exists a *t*_0_ such that *S*(*t*) ≤ *y*(*t*) ≤ *A*[*μ*(1 − *p*)+(*ω* + *τ*)]/*μ*(*μ* + *ω*) + *ε* as *t* > *t*_0_.

Considering the Lyapunov function *L* = *σE* + (*μ* + *σ*)*I*, thus

(55) L′=σE′+(μ+σ)I′=I[σβS(1+αI)−(μ+σ)(μ+τ+ϵ)].

For *R*_0_(1 + *αA*/*μ*) < 1, we can choose *ε* small enough such that (1 + *αA*/*μ*)*R*_0_ − 1 + (1 + *αA*/*μ*)*εσβ*/(*μ* + *σ*)(*μ* + *τ* + *ϵ*) < 0. Thus,

(56) L′≤σβAμ1−p+ω+τμμ+ω+εmmc·1+αAμ−(μ+σ)(μ+τ+ϵ)I=(μ+σ)(μ+τ+ϵ) ·[(1+αAμ)R0−1+(1+αA/μ)εσβ(μ+σ)(μ+τ+ϵ)]I≤0,

and *L*′ = 0 if and only if *I* = 0. The singleton *P*_0_ is the maximum positive invariant set in {(*S*, *V*, *E*, *I*) ∈ *Ω*, *L*′ = 0}. The global stability of *P*_0_ for every solution follows from LaSalle's Invariance Principle.

If *R*_1_^*^ < 1, system (3) has two endemic equilibrium when *R*_0_^*^ < *R*_0_ < 1. Furthermore, system (3) shows a backward bifurcation. That means we should require that *R*_0_ becomes much smaller than unity (less than a critical value *R*_0_^*^) so that the disease can be eliminated. Thus, when *R*_1_^*^ < 1 the disease-free equilibrium *P*_0_ is globally asymptotically stable in *Ω* when *R*_0_ < *R*_0_^*^.

For system (3), we discuss global stability of the endemic equilibrium *P* ^*^ for *R*_0_ > 1. Due to *S* + *V* + *E* + *I* → *A*/*μ* when *t* → *∞*, we can determine *V*(*t*) by *S*(*t*), *E*(*t*), and *I*(*t*). So system (3) can be changed into the following limit system:

(57) S˙t=A(1−p)+ω(Aμ−S(t)−E(t)−I(t)) +τI−μS−βSI(1+αI),E˙(t)=βSI(1+αI)−μE−σE,I˙(t)=σE−μI−τI−ϵI.

We need the following results [15] to obtain the result we want.

Consider the system as follows:

(58) dxdt=f(t,x),

(59) dydt=g(y),

where *f* and *g* are locally Lipschitz in *x* ∈ *R* ^*n*^ and continuous. And for all positive *t* values its solutions exist. If *f*(*t*, *x*) → *g*(*x*) when *t* → *∞* locally uniformly for *x* ∈ *R* ^*n*^, then system (58) is defined as asymptotically autonomous with limit system (59).

**Lemma 11 .**

Set *P* is a locally asymptotically stable equilibrium of (59) and *x* is the *ω*-limit set of a forward bounded solution *x*(*t*) of (58). If *x* includes a point *y*_0_ such that the solution of (59) with *y*(0) = *y*_0_ converges to *P* when *t* → *∞*, then *ω* = *P*; that is, *x*(*t*) → *P* when *t* → *∞*.

**Corollary 12 .**

If solutions of system (58) are bounded and the equilibrium *P* of the limit system (59) is globally asymptotically stable, then any solution *x*(*t*) of system (58) satisfies *x*(*t*) → *P* when *t* → *∞*.

Next, we obtain sufficient conditions that endemic equilibrium *P* ^*^ is globally asymptotically stable for *R*_0_ > 1 by the geometrical approach [9]. Firstly, we briefly introduce this geometrical approach.

Let a *C* ^1^ function *x* → *f*(*x*) ∈ *R* ^*n*^ be in an open set *D* ∈ *R* ^*n*^. Consider the differential equation

(60) $$\begin{array}{c} \frac{dx}{dt}=f\left(x\right). \end{array}$$

Denote *x*(0, *x*_0_) = *x*_0_ by *x*(*t*, *x*_0_) which is the solution to (60). We establish the following two assumptions.

(H1) There exists a compact absorbing set *K* ⊂ *D*.
(H2) Equation (60) has a unique equilibrium $\overset{-}{x}$ in *D*.

If the equilibrium $\overset{-}{x}$ is locally stable, it is globally stable in *D* and all trajectories in *D* converge to $\overset{-}{x}$. For *n* ≥ 2, we mean a condition satisfied by *f* which rules out the existence of nonconstant periodic solutions of (60) by Bendixson's criterion. The classical Bendixson's condition div*f*(*x*) < 0 for *n* = 2 is robust under *C* ^1^ local perturbations of *f*. About higher-dimensional systems, the *C* ^1^ robust properties have been discussed.

If there exists a neighborhood *U* of *x*_0_ and *T* > 0 such that *U*∩*x*(*t*, *U*) is empty for all *t* > *T*, then a point *x*_0_ ∈ *D* is called wandering for (59). For example, all limit points and equilibria are nonwandering. We will introduce the global stability principle in [19] which is suited for autonomous systems.

**Lemma 13 (see [19]).**

Assume that (*H*1) and (*H*2) hold. And suppose that (60) satisfies Bendixson's criterion that is robust under *C* ^1^ local perturbations of *f* at all nonequilibrium nonwandering points for (60). Then, $\overset{-}{x}$ is globally stable in *D* provided it is stable.

To have the robustness required by Lemma 13, we show the following Bendixson criterion [19]. Let *x* → *P*(*x*) be a matrix-valued function that is *C* ^1^ for *x* ∈ *D*. Assume that *P* ^−1^(*x*) exists and is continuous for *x* ∈ *K*, which is the compact absorbing set. Define a quantity $\overset{-}{q_{2}}$ as

(61) $$\begin{array}{c} \overset{-}{q_{2}}=\underset{t\to \infty }{\lim}\sup\;\underset{x\in K}{\sup}\frac{1}{t}\overset{t}{\underset{0}{\int }}\mu \left(B\left(x\left(s,x_{0}\right)\right)\right)ds, \end{array}$$

where

(62) $$\begin{array}{c} B=P_{f}P^{-1}+P\frac{\partial f^{\left[2\right]}}{\partial x}P^{-1}. \end{array}$$

By substituting the derivative in the direction of *f* into each entry *p* of *P*, the matrix *P*_*f*_ is obtained. *μ*(*B*) is the Lozinski$\overset{˘}{1}$ measure of *B* in terms of a vector norm |·| in *R* ^*N*^:

(63) $$\begin{array}{c} \mu \left(B\right)=\underset{h\to 0^{+}}{\lim}\frac{\left|I+hB\right|-1}{h}. \end{array}$$

If *D* is simply connected, the condition $\overset{-}{q_{2}}<0$ excludes the existence of any orbit that attracts a simple closed rectifiable curve that is invariant for (62), such as homoclinic orbits, heteroclinic cycles, and periodic orbits in [19]. And it is robust under *C* ^1^ local perturbations of *f* near any nonequilibrium point that is nonwandering. In particular, the following lemma is proved in [19].

**Lemma 14 .**

Assume that *D* is simply connected and that the hypotheses (*H*1) and (*H*2) hold. Then, if  $\overset{-}{q_{2}}<0$, the unique equilibrium $\overset{-}{x}$ of (62) is globally stable in *D*.

Next, we will obtain the main result.

**Theorem 15 .**

If *R*_0_ > 1, system (3) admits a unique endemic equilibrium *P* ^*^. It is globally asymptotically stable in terms of solutions of (3) initiating in the interior of *Ω*, provided that inequality (77) or (78), and *c*_1_ > 0, *c*_1_ *c*_2_ − *c*_3_ > 0 are satisfied.

**Proof**

The Jacobian of system (57) is as follows:

(64) J=−μ−ω−βI(1+αI)−ω−ω+τ−βS(1+2αI)βI(1+αI)−μ−σβS(1+2αI)0σ−τ−μ−ϵ.

From Theorem 3(ii), we obtain that there exists the endemic equilibrium *P* ^*^ and it is unique due to *R*_0_ > 1. We will analyse the stability of *P* ^*^ by the method in [9]. Due to Lemma 14, the global stability of *P* ^*^ requires the following sufficient conditions: (i) there must exist a compact absorbing set in the interior of *Ω* (i.e., condition (H1)); (ii) *P* ^*^ in the interior of *Ω* is unique (i.e., condition (H2)); and (iii) the requirement $\overset{-}{q_{2}}<0$.

System (3) satisfies (H1)-(H2) under the assumption *R*_0_ > 1. Actually, *P*_0_ is unstable when *R*_0_ > 1. As *P*_0_ is unstable and *P*_0_ ∈ ∂*Ω*, we can obtain the uniform persistence.

As *Ω* is bounded, the uniform persistence implies that there exist a compact absorbing set in the interion of *Ω* for system (3) (see [20]). Therefore, (H1) is verified. Also, *P* ^*^ is the only equilibrium in the interior of *Ω*, so that *P* ^*^ is unique; that is, (H2) is verified, too.

Next we will look for conditions which satisfied (H3). Consider the Jacobian matrix (64) and get the second additive compound matrix *J* ^[2]^(*S*, *E*, *I*):

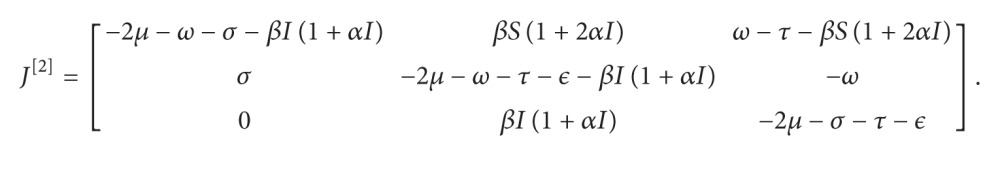
(65)


Let *p*(*x*) = *P*(*S*, *E*, *I*) = diag{*E*/*I*, *E*/*I*, *E*/*I*}. Then *P*_*f*_ *P* ^−1^ = diag{*E*′/*E* − *I*′/*I*, *E*′/*E* − *I*′/*I*, *E*′/*E* − *I*′/*I*}. Thus, the matrix *B* = *P*_*f*_ *P* ^−1^ + *PJ* ^[2]^ *P* ^−1^ can be written in block form as

(66) $$\begin{array}{c} B=\left[\begin{array}{cc} B_{11} & B_{12}\\ B_{21} & B_{22} \end{array}\right], \end{array}$$

where

(67) $$\begin{array}{c} B_{11}=\frac{E^{\prime }}{E}-\frac{I^{\prime }}{I}-\beta I\left(1+\alpha I\right)-2\mu -\omega -\sigma ,\\ B_{12}=\left[\beta S\left(1+2\alpha I\right),\beta S\left(1+2\alpha I\right)+\omega -\tau \right],\\ B_{21}={\left[\sigma ,0\right]}^{T},\\ B_{22}=\left[\begin{array}{cc} \frac{E^{\prime }}{E}-\frac{I^{\prime }}{I}-\beta I\left(1+\alpha I\right)-2\mu -\omega -\tau -\epsilon & -\omega \\ \beta I\left(1+\alpha I\right) & \frac{E^{\prime }}{E}-\frac{I^{\prime }}{I}-2\mu -\sigma -\tau -\epsilon \end{array}\right]. \end{array}$$

Set $\left(\overset{-}{k},\overset{-}{l},\overset{-}{m}\right)$ be the vectors in *R* ^3^. We choose a standard in *R* ^3^ as $\left|\left(\overset{-}{k},\overset{-}{l},\overset{-}{m}\right)\right|=\max\{\left|\overset{-}{k},\overset{-}{l}+\overset{-}{m}\right|\}$ and set *μ* be the Lozinski$\overset{˘}{1}$ measure in term of this standard. Applying the technique in [21], the following can be obtained:

(68) $$\begin{array}{c} \mu \left(B\right)\leq \sup\left\{g_{1},g_{2}\right\}, \end{array}$$

where

(69) $$\begin{array}{c} g_{1}=\mu_{1}\left(B_{11}\right)+\left|B_{12}\right|,\;\;g_{2}=\mu_{1}\left(B_{22}\right)+\left|B_{21}\right|. \end{array}$$

We can obtain

(70) μ1(B11)=B11=E′E−I′I−βI(1+αI)−2μ−ω−σ,B21=σ,μ1B22=max⁡⁡E′E−I′I−2μ−ω−τ−ϵ,mmmmccE′E−I′I−2μ−σ−τ−ϵ−ω=E′E−I′I−2μ−ω−τ−ϵ,B12=max⁡{βS(1+2αI),βS(1+2αI)+ω−τ}=ω−τ+βS1+2αI,ω−τ>0,βS1+2αI,ω−τ<0.

From the second and third equations of system (57), we can obtain

(71) E′E=βSI(1+αI)E−(μ+σ),I′I=σEI−(μ+τ+ϵ).

Thus, we obtain

(72) g1=μ1(B11)+B12=E′E−I′I−βI(1+αI)−2μ−σ + βS1+2αI−τ,ω>τ,E′E−I′I−βI1+αI−2μ−ω−σ + βS1+2αI,ω<τ,g2=μ1(B22)+B21=E′E−I′I−2μ−ω−τ−ϵ+σ.

We can get

(73) $$\begin{array}{c} g_{1}\leq \left\{\begin{array}{cc} \frac{E^{\prime }}{E}-\mu -\sigma +\epsilon -\beta I\left(1+\alpha I\right) & \\ \;+\;\beta S\left(1+2\alpha I\right), & \omega >\tau ,\\ \frac{E^{\prime }}{E}-\mu -\omega -\sigma +\epsilon -\beta I\left(1+\alpha I\right) & \\ \;+\;\beta S\left(1+2\alpha I\right), & \omega <\tau , \end{array}\right.\\ g_{2}\leq \frac{E^{\prime }}{E}-\mu -\omega +\sigma . \end{array}$$

Hence

(74) μ(B)≤E′E+max⁡−μ−ω+σ,mmmmmm−μ−σ+ϵmmmmmm−βI(1+αI)mmmmmm+βS(1+2αI),ω>τ,E′E+max⁡−μ−ω+σ,mmmmmm−μ−ω−σ+ϵmmmmmm−βI(1+αI)mmmmmm+βS(1+2αI),ω<τ.

Considering *c* ≤ *S*, *I* ≤ *A*/*μ*, where *c* is the constant of uniform persistence; it is obvious that

(75) $$\begin{array}{c} \mu \left(B\right)\leq \frac{E^{\prime }}{E}-d, \end{array}$$

where

(76) d=min⁡−μ−ω+σ,Aμmmmc−μ−σ+ϵ−βc(1+αc)mmmc+βAμ(1+2αAμ),ω>τ,min⁡−μ−ω+σ,Aμmmmc−μ−ω−σ+ϵ−βc(1+αc)mmmc+βAμ(1+2αAμ),ω<τ.

And if

(77) $$\begin{array}{c} \mu +\omega <\sigma ,\\ \beta c\left(1+\alpha c\right)+\mu +\sigma -\epsilon <\beta \frac{A}{\mu }\left(1+2\alpha \frac{A}{\mu }\right),\;\omega >\tau , \end{array}$$

or

(78) $$\begin{array}{c} \mu +\omega <\sigma ,\\ \beta c\left(1+\alpha c\right)+\mu +\omega +\sigma -\epsilon <\beta \frac{A}{\mu }\left(1+2\alpha \frac{A}{\mu }\right),\;\omega <\tau , \end{array}$$

holds, then *d* > 0.

For each (*S*(*t*), *E*(*t*), *I*(*t*)) ∈ *Ω*, we obtain

(79) 1t∫0tμ(B)ds≤1t∫0t1μ(B)ds+1t∫t1tμ(B)ds≤1t∫0t1μ(B)ds+1tlog⁡E(t)E(t1)−d≤−d2<0.

Due to Theorem 4 and Lemma 14, if *R*_0_ > 1, then the endemic equilibrium *P* ^*^ of system (3) is globally stable in *Ω*.

## 6. Numerical Simulations

Next, we show some numerical examples to support our analytic results.

**Example 1 .**

**Figure 1:**
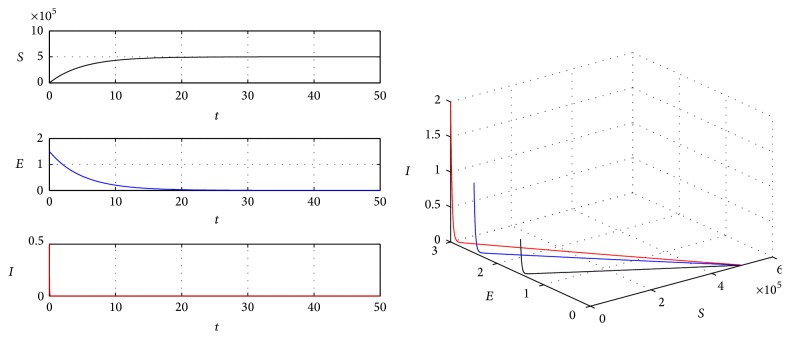
The global stability of disease-free equilibrium.

We take parameters *p* = 0.9, *A* = 1000000, *ω* = 0.000005, *μ* = 2, *τ* = 40, *β* = 0.00009, *α* = 0.000005, *σ* = 0.006, and *ϵ* = 0.08. Then we can obtain *M* = 168.8254 > *στ*(*μ* + *ω*) = 0.4800 and *R*_0_ = 0.00031987 < 1 which satisfies Theorem 2 and *R*_0_(1 + *αA*/*μ*) = 0.0011 < 1 which satisfies Theorem 10. Therefore, system (3) has a disease-free equilibrium *P*_0_(50001,450000,0, 0) and it is globally asymptotically stable (Figure 1).

**Example 2 .**

**Figure 2:**
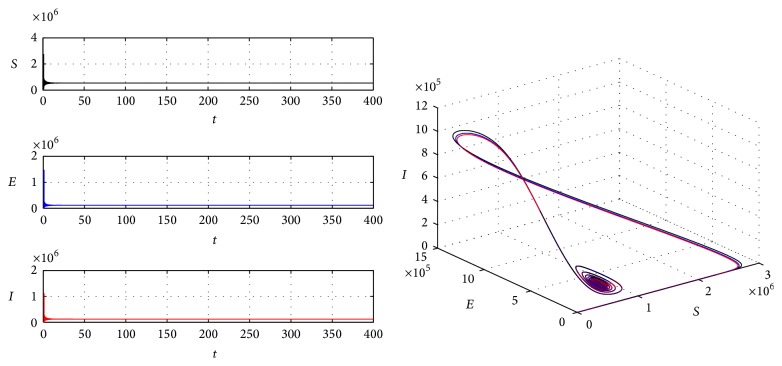
The global stability of endemic equilibrium.

**Figure 3:**
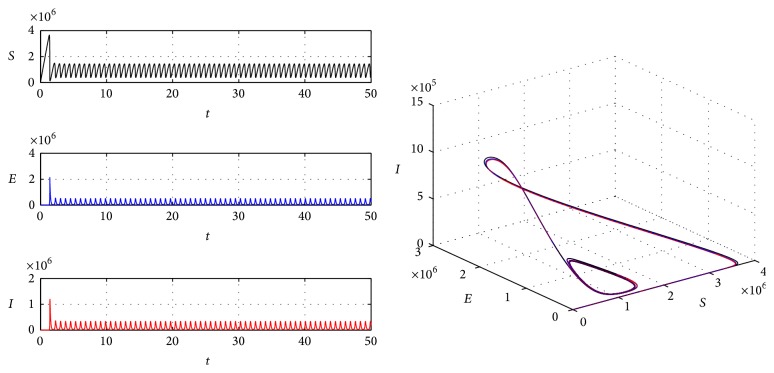
The periodic solution of system (3).

We take parameters *p* = 0.6, *A* = 1000000, *β* = 0.00005, *α* = 0.000005, *μ* = 0.02, *ω* = 0.05, *σ* = 45.6, and *ϵ* = 23. Under these parameters, due to Theorem 9, we calculate the critical value *τ* ^*^ = 39.9918. If we take *τ* = 20 < *τ* ^*^, we can get *M* = 137.3801 > *στ*(*μ* + *ω*) = 63.8400, *R*_0_ = 48.1293, *R*_1_^*^ = 267.6519, and *R*_0_^*^ = 0.0032. Therefore *R*_0_^*^ < 1 < *R*_0_ < *R*_1_^*^, due to Theorem 3 system (3) has a disease-free equilibrium *P*_0_(41429000,8571400,0, 0) and an endemic equilibrium *P* ^*^(432250,187060,198280,8571400). And we can calculate *d* = 45.53 > 0 which guarantees $\overset{-}{q_{2}}<0$. From Theorem 15, we can get that the endemic equilibrium is globally asymptotically stable, which is demonstrated by Figure 2. If we take *τ* = 40 > *τ* ^*^, then the endemic equilibrium *P* ^*^ becomes unstable and a periodic orbits bifurcates from *P* ^*^, which is demonstrated by Figure 3.

## 7. Discussion

In this paper, considering disease-caused death and partial permanent immunity, we modified the SEIV epidemic model in [15]. Applying the method of [16], we calculated the basic reproduction number *R*_0_ and found that when *R*_0_ = 1 and *R*_1_^*^ < 1 system (3) shows backward bifurcation. If *R*_1_^*^ < 1, system (3) has a unique endemic equilibrium when *R*_0_ ≥ 1 and has two endemic equilibria when *R*_0_ < 1. If *R*_1_^*^ > 1, system (3) has a unique endemic equilibrium when *R*_0_ ≥ 1 and has no endemic equilibrium when *R*_0_ < 1. Also system (3) always has a disease-free equilibrium *P*_0_. Local and global asymptotic stability of the disease-free equilibrium are determined by *R*_0_ < 1 and *R*_0_(1 + *αA*/*μ*) < 1, respectively. Also we have studied the local and global asymptotic stability of the endemic equilibrium. Moreover, taking the disease-caused death rate *τ* as bifurcation parameter, we discussed the Hopf bifurcation of system (3). We found that when *R*_0_ > 1, there is always a critical value *τ* ^*^, such that system (3) exhibits Hopf bifurcation at *P* ^*^ when *τ* passes through *τ* ^*^.

From the sense of epidemiology, when *R*_0_(1 + *αA*/*μ*) < 1, if *R*_1_^*^ > 1 holds or *R*_1_^*^ < 1, *R*_0_ < *R*_0_^*^ hold; system (3) has one disease-free equilibrium which is globally stable. Namely, the disease will be eradicated. And when *R*_0_ > 1 and inequality (77) or (78) holds, system (3) has a unique endemic equilibrium *P* ^*^ which is global asymptotically stable. Under this circumstance, the infectious disease becomes endemic disease. If *R*_0_ > 1, system (3) has a unique endemic equilibrium *P* ^*^ and we found that when the rate *τ* becomes sufficiently large the disease will break out periodically. And differentiating the bifurcation coefficient *a* partially with respect to *p*, we can get ∂*a*/∂*p* = −*σαAμ*(*μ* + *σ*) < 0, which means that vaccinating more susceptible populations decreases the likelihood of the occurrence of backward bifurcation [15].
